# Late-onset spastic ataxia phenotype in a patient with a homozygous *DDHD2* mutation

**DOI:** 10.1038/srep07132

**Published:** 2014-11-24

**Authors:** Hiroshi Doi, Masao Ushiyama, Takashi Baba, Katsuko Tani, Masaaki Shiina, Kazuhiro Ogata, Satoko Miyatake, Yoko Fukuda-Yuzawa, Shoji Tsuji, Mitsuko Nakashima, Yoshinori Tsurusaki, Noriko Miyake, Hirotomo Saitsu, Shu-ichi Ikeda, Fumiaki Tanaka, Naomichi Matsumoto, Kunihiro Yoshida

**Affiliations:** 1 Department of Neurology and Stroke Medicine, Graduate School of Medicine, Yokohama City University, 3-9 Fukuura, Kanazawa-ku, Yokohama 236-0004, Japan; 2 Department of Human Genetics, Graduate School of Medicine, Yokohama City University, 3-9 Fukuura, Kanazawa-ku, Yokohama 236-0004, Japan; 3 Department of Neurology, Kenwakai Hospital, 1936 Kanaenakadaira, Iida 395-8522, Japan; 4 School of Life Sciences, Tokyo University of Pharmacy and Life Sciences, 1432-1 Horinouchi, Hachioji, Tokyo, 192-0392, Japan; 5 Department of Biochemistry, Graduate School of Medicine, Yokohama City University, 3-9 Fukuura, Kanazawa-ku, Yokohama 236-0004, Japan; 6 Department of Neurology, Graduate School of Medicine, The University of Tokyo, 7-3-1 Hongo, Bunkyo-ku, Tokyo, 113-8655, Japan; 7 Genomics Division, MS 84-171, Lawrence Berkeley National Laboratory, Berkeley, CA 94720, USA; 8 Department of Medicine (Neurology & Rheumatology), Shinshu University School of Medicine, 3-1-1 Asahi, Matsumoto 390-8621, Japan; 9 Division of Neurogenetics, Department of Brain Disease Research, Shinshu University School of Medicine, 3-1-1 Asahi, Matsumoto 390-8621, Japan

## Abstract

Autosomal recessive cerebellar ataxias and autosomal recessive hereditary spastic paraplegias (ARHSPs) are clinically and genetically heterogeneous neurological disorders. Herein we describe Japanese siblings with a midlife-onset, slowly progressive type of cerebellar ataxia and spastic paraplegia, without intellectual disability. Using whole exome sequencing, we identified a homozygous missense mutation in *DDHD2*, whose mutations were recently identified as the cause of early-onset ARHSP with intellectual disability. Brain MRI of the patient showed a thin corpus callosum. Cerebral proton magnetic resonance spectroscopy revealed an abnormal lipid peak in the basal ganglia, which has been reported as the hallmark of *DDHD2*-related ARHSP (SPG 54). The mutation caused a marked reduction of phospholipase A_1_ activity, supporting that this mutation is the cause of SPG54. Our cases indicate that the possibility of SPG54 should also be considered when patients show a combination of adult-onset spastic ataxia and a thin corpus callosum. Magnetic resonance spectroscopy may be helpful in the differential diagnosis of patients with spastic ataxia phenotype.

Autosomal recessive cerebellar ataxias (ARCAs) and autosomal recessive hereditary spastic paraplegias (ARHSPs) are clinically and genetically heterogeneous neurological disorders. Mutations in more than 30 different genes have been identified for ARCAs, and those of 38 different genes have been identified for ARHSPs. Genetic diagnosis of ARCAs and ARHSPs has been difficult, because of genetic and clinical heterogeneity. In addition, the accompanying symptoms can vary even within the same disease (e.g. spastic paraplegia 7 (SPG7) can present with both a pure and complex phenotype^1^), making correct diagnosis more difficult. Recent advances in parallel sequencing technology are making a major contribution, not only to the identification of novel responsible genes^2^, but also to finding genetic causes even in unrecognized phenotypes^3^, and to the comprehensive genetic diagnosis of ARCAs and ARHSPs^4^,^5^. Herein, we describe Japanese siblings exhibiting a midlife-onset spastic ataxia with a novel homozygous *DDHD2* mutation found by exome-sequencing.

## Results

**Figure 1:**
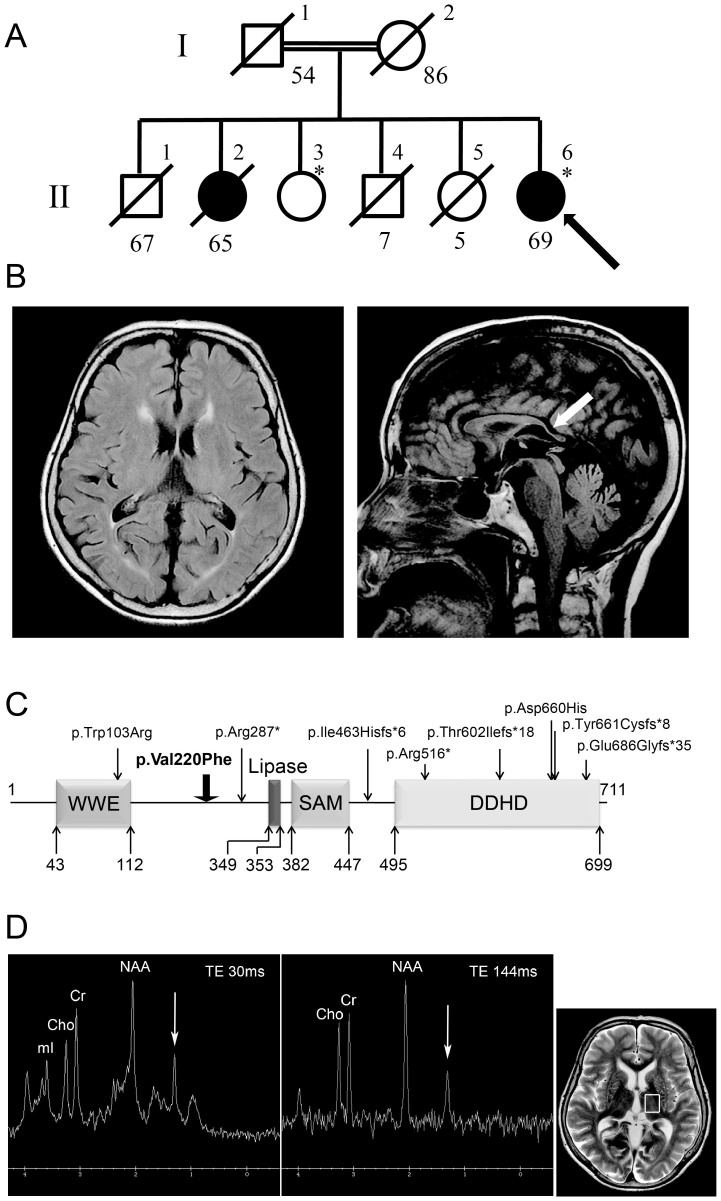
Familial pedigree, brain MRI and proton MRS of a patient with homozygous *DDHD2* mutation. (A): Familial pedigree. * indicates members whose genomic DNA was available for this study (II-3 and II-6). Arrow indicates the proband (II-6). Homozygosity mapping and linkage analysis were performed using DNA from the proband and the unaffected sibling (II-3). (B): Brain MRI of II-6 at 69 years of age. Axial and sagittal sections of fluid-attenuated inversion recovery image are shown. Mild atrophy of the cerebellum and the thinness of the splenium of the corpus callosum (arrow) are observed. (C): Schematic presentation of DDHD2 and mutations. The thick arrow indicates the location of the mutation in the patient. (D): Proton MRS obtained from left thalamus, at a magnetic field of 3 Tesla (echo time 30 ms and 144 ms, respectively). Arrows indicate the pathologic lipid peak at 1.3 ppm. mI: myo-inositol, Cho: choline, Cr: creatine, NAA: N-acetylaspartate.

**Figure 2:**
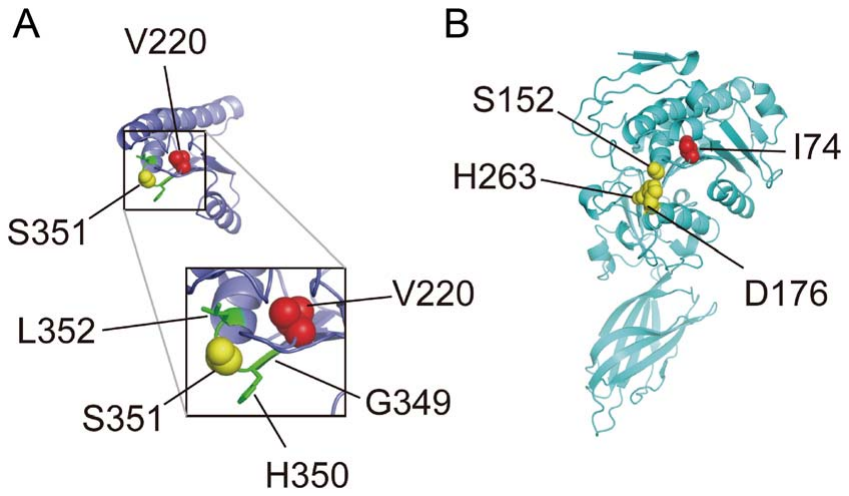
Structural consideration of an impact of the p.Val220Phe mutation in human DDHD2. (A–B): A modeled structure of the region around Val220 in human DDHD2 (A) and the crystal structure of the catalytic domain of GPLRP2 chimerized with the C-terminal domain of HPL (PDB id; 1GPL) (B) are shown. The side chains of Val220 in A and the corresponding residue Ile74 in B are shown in red spheres. The catalytic triad in B and a component residue of the triad, Ser351, in A are shown in yellow spheres^28^. In A, a stretch of the sequence, Gly^349^-His-Ser-Leu^352^, which is conserved in most lipases^22^, is colored green, and their side chains were shown as sticks, except for Ser351. Inset: close-up view of the squared region.

**Figure 3:**
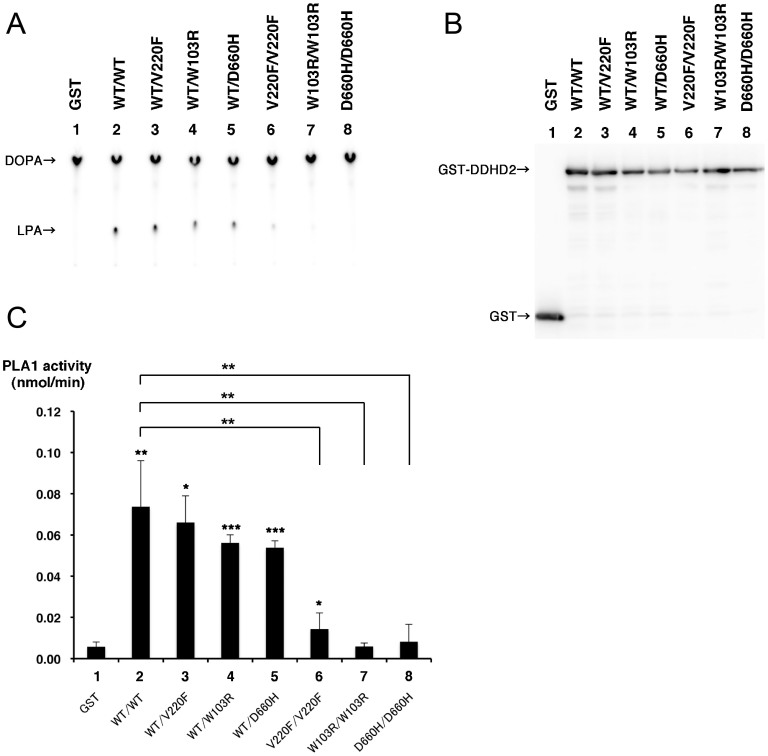
PLA_1_ activity of DDHD2 mutants. GST and GST-tagged DDHD2 WT and/or DDHD2 mutants were expressed in 293T cells and partially purified. Their PLA_1_ activities were measured using ^32^P-labeled DOPA as a substrate, as described in “Methods”. The reaction products were analyzed by TLC. (A): Representative results of TLC analysis. GST (lane 1), GST-DDHD2 WT (lane 2), a mixture of GST-DDHD2 WT and one of GST-DDHD2 mutants (p.Val220Phe (lane 3), p.Trp103Arg (lane 4), and p.Asp660His (lane 5)), and GST-DDHD2 mutants alone (p.Val220Phe (lane 6), p.Trp103Arg (lane 7), and p.Asp660His (lane 8)) were used. Positions of DOPA and LPA are indicated by arrows. The product LPA was clearly detected in the lane with GST-DDHD2 WT but not with GST alone. The amounts of LPA were markedly reduced in the cases of all the three mutants. (B): A representative image of Western blotting analysis. GST and GST fusion proteins used for the above PLA_1_ assay were analyzed by Western blotting using an anti-GST antibody. One-fifth of amount of each sample used in A was loaded. Lane numbers are the same as in A. (C): Comparison of PLA_1_ activity. The intensities of spots on a TLC plate were quantified using Multi Gauge V3.0 software (Fujifilm). The PLA_1_ activity (an amount (nmol) of LPA formed per min) was calculated from the intensities of LPA and DOPA spots. Data are shown as means ± S.D. from 3 or more independent experiments. Numbers in the graph are the same as in A. The asterisks indicate statistically significant differences between the DDHD2 mutants and control DDHD2 WT (asterisks placed above each bracket), or between GST and each condition of DDHD2 (asterisks placed above each bar) (*P < 0.05, **P < 0.001 and ***P < 0.0005, Student's t-test).

Among the children of first-cousin parents, two sisters were affected (Figure 1A). The early developmental milestones of the proband (II-6) were normal. At the age of approximately 45 years old, she developed gait unsteadiness and dysarthria. She was 69 years old at the last examination, and could not stand without holding on to something. She had gaze-evoked horizontal nystagmus, dysarthria, extensor plantar reflexes, mild limb ataxia, moderate truncal ataxia, postural tremor in the head and upper extremities, decline of vibratory sense in the lower extremities, and urinary incontinence. Patellar tendon reflexes were increased, while Achilles tendon reflexes were absent. Cognitive impairments including callosal apraxia were not observed. Laboratory biochemistry results were normal, including serum liver enzymes, ammonia, thyroid hormones, copper, α-fetoprotein, vitamin E and very long-chain fatty acids. Serum antibody for Human T lymphotropic virus type 1 was negative. Galactocerebrosidase activity in leukocytes was normal. Blood amino-acid analysis and urinary organic acid analysis revealed no apparent deviance. A nerve conduction study disclosed a mild slowing of motor and sensory nerve conduction velocities (between 36.0 and 46.3 m/s) with reduced compound muscle action potentials. Brain magnetic resonance imaging revealed mild atrophy of the cerebellum, and a thinness of the splenium of the corpus callosum (Figure 1B). Neither atrophy nor cross sign were observed in the brainstem. The patient was negative for the genetic alterations associated with spinocerebellar ataxia (SCA)1, SCA2, SCA3, SCA6, SCA7, SCA12, SCA17 and dentatorubral pallidoluysian atrophy. Her elder sister (II-2) developed gait unsteadiness at the age of 38 years, and by age 55 years, could not walk independently. She showed saccadic eye pursuit, dysarthria, dysphagia, limb muscle weakness, extensor plantar reflexes, limb and truncal ataxia, and urinary incontinence. She died at 65, most likely because of hepatic encephalopathy with hyperammonemia (164 μg/dl, normal range 12–66 μg/dl), high serum lactate level (22.9 mg/dl, normal range 4–16 mg/dl) and pyruvate (2.3 mg/dl, normal range 0.3–0.9 mg/dl), and ketonuria. Serum copper and ceruloplasmin were normal. Eventually, a precise cause of hepatic insufficiency could not be detected. Another two siblings (II-4 and II-5) died at early ages (7 and 5 years, respectively) with unknown cause, but it is unlikely that the causes of their deaths were related to spastic ataxia, considering that they died in childhood. Homozygosity mapping and linkage analysis identified 11 candidate regions totalized to ~240 Mb, with the maximum LOD score equaling 1.32 (Table S2). When an adult patient exhibits both cerebellar ataxia and spasticity, the primary diagnostic considerations are autosomal recessive ataxia of Charlevoix-Saguenay, late-onset Friedreich ataxia or SPG7^6^. However, none of the related genes, *SACS*, *FRDA* and *SPG7*, were located in the candidate regions of our patient. As a result of whole exome-sequencing of the proband, approximately 39.8 million paired-reads were mapped to the human reference genome. A coverage analysis revealed that 95.9% of the bases within the target regions were covered by 10 reads or more. In total, 37,553 variations, which were unregistered in dbSNP137 and registered as uncommon SNPs with minor allele frequency <1%, were detected. Among these, 2,986 variations (including 1,148 homozygous variants) were located in exons or splice sites (within 2 bp of the boundaries). Only the eight homozygous missense single nucleotide variations (SNVs) remained in the ~240-Mb candidate regions with the frequency <1% in exome data from 575 “in house” Japanese controls (Table 1). Sanger sequencing confirmed that all of these SNVs were homozygous in the proband and heterozygous in the unaffected sibling. Among the SNVs, the c.658G > T [p.Val220Phe] of *DDHD2* (Figure 1C) was of interest, because mutations of *DDHD1* and *DDHD2*, which code for members of the intracellular phospholipase A_1_ (PLA_1_), have recently been found to be the causative genes for ARHSPs (SPG28 and SPG54)^7^,^8^. Furthermore, only the SNVs of *DDHD2* and *FAM222A* were consistently predicted to be disruptive in protein function when analyzed with multiple tools including Polyphen2, SIFT and Mutation Taster^9^,^10^,^11^, while the predictions for the other six SNVs were benign or inconclusive (Table 1). Considering the allele frequency of *FAM222A* SNV in Japanese control exome data (4/575), it is unlikely that the SNV is the cause of extremely rare diseases. The potentially compound heterozygous SNVs detected in the proband are listed, indicating that none of the listed genes is likely to be the cause of the disease (Table S3). We further checked whether any other causative variations were present in known ARCA or ARHSP genes, which are listed in Table S1. We confirmed that no pathological homozygous or compound heterozygous SNVs were found in these genes. Patients with *DDHD2* mutations have been reported to show very early-onset (before the age of 6 years) spastic paraplegia with intellectual disability (SPG54), occasionally associated with strabismus and/or hypoplasia of the optic nerve (Table 2)^8^,^12^,^13^,^14^. Brain MRIs of these patients showed a thin corpus callosum with periventricular white-matter hyperintensity^8^. As a unique finding, proton magnetic resonance spectroscopy (^1^H-MRS) revealed an abnormal lipid peak in the basal ganglia and thalamus area (Figure 1D). Considering the highly characteristic ^1^H-MRS findings, and the observations that the patient carried a novel homozygous p.Val220Phe of *DDHD2* predicted as deleterious^15^,^16^ (and not present in 575 Japanese controls by whole exome sequencing or 429 Japanese controls by Sanger sequencing), we thought that the *DDHD2* mutation was the causative agent in this patient. Because most causative mutations of SPG54 were protein-truncating (Figure 1C), loss of DDHD2 function is plausible. We first checked intracellular distribution of p.Val220Phe and wild type (WT) in HEK293T cells, but found no difference (Figure S1). The result indicated that p.Val220Phe does not severely affect the conformation or stabilities of DDHD2. We then assessed an impact of the p.Val220Phe mutation by mapping the mutation on a 3D structure. Val220 is predicted to be involved in a hydrophobic core near the candidate catalytic site, suggesting that the p.Val220Phe mutation may impair lipase activity (Figure 2). Although SPG54-linked point mutations were reported (Table 2), whether the mutations affect enzymatic activity was not examined. We thus analyzed the PLA_1_ activity of the p.Val220Phe mutant as well as p.Trp103Arg and p.Asp660His mutants, both of which were reported to be linked to SPG54^14^. The results clearly demonstrated that the p.Val220Phe mutant as well as both the p.Trp103Arg and p.Asp660His mutants has a statistically significant reduction in their PLA_1_ activity (Figure 3A–C, lane 6–8). Notably, the p.Val220Phe mutant, but not other two mutants still retained a marginal PLA_1_ activity (Figure 3A–C, lane 6). We also co-transfected equal amounts of the WT- and each mutant-expressing plasmids, and then measured PLA_1_ activity. These conditions mimicked the heterozygous states of healthy carriers with both wild-type and mutant alleles. In these conditions, the PLA_1_ activity was not severely affected (Figure 3A–C, lane 3–5), indicating that all of the mutants did not have a dominant-negative effect on the WT DDHD2. These results strongly indicated that the p.Val220Phe mutation of *DDHD2* was indeed a culprit mutation in this patient.

## Discussion

Compared with the clinical presentations described in previous reports, our patients were quite a bit older at onset, the main phenotype was spastic ataxia, and intellectual disability was not observed. Mild polyneuropathy was observed in our case, revealing the phenotypic variability of SPG54. One of our patients showing spastic ataxia phenotype (II-2) developed hepatic encephalopathy with unknown cause. Because we could not detect the evidence of metabolic disease in the proband, whether hepatic encephalopathy was a part of the SPG54 is unknown. It is possible that her sibling (II-2), but not the proband, might have had some metabolic problems other than spastic ataxia, because she did not have apparent evidence of acquired liver diseases (such as viruses or toxic agents) or portacaval shunt. Brain MRIs of our case showed mild white-matter hyperintensity and a thin corpus callosum, sharing these common characteristics with previous cases. In addition, mild cerebellar atrophy was recognized. In diseases presenting with SCA or SPG, abnormal ^1^H-MRS findings have been reported, especially when the phenotype is the consequence of metabolic disorders. In these cases, some with white matter changes have shown increased myo-inositol/creatine (Cr) and decreased N-acetylaspartate/Cr, reflecting astrogliosis and neuroaxonal loss^17^,^18^. Although broad lipid peaks have sometimes been detected in child food adrenoleukodystrophy and peroxisome disorders, emergence of clear, sharp lipid peaks have been reported in very limited diseases such as Sjögren-Larsson syndrome^19^,^20^ hypomyelination and congenital cataract^21^, which show distinct clinical manifestations from SCA and SPGs. No SNVs were detected in the causative gene of Sjögren-Larsson syndrome (*ALDH3A2*). Because a sharp lipid peak has not been reported in any other SCA or SPGs, the finding could be considered as highly characteristic for SPG54^8^. However, mutations in a group of genes involved in lipid metabolism including *CYP7B1* (SPG5), *DDHD1* (SPG28), *FA2H* (SPG35), *PNPLA6* (SPG39), *GBA2* (SPG46) and *CYP2U1* (SPG56) have been found as the causes of ARHSPs. The ^1^H-MRS findings of the ARHSPs remain to be investigated. DDHD2 is a member of intracellular PLA_1_^22^, which hydrolyzes an acyl group from phospholipids at the *sn*-1 position. The tandem SAM-DDHD domain of DDHD2 is essential for binding to phosphatidylinositol phosphate^23^. Most *DDHD2* mutations reported reside in the SAM-DDHD domain (Figure 1C), but it has been never assessed whether the mutations actually impair the PLA_1_ activity. Previously, only two missense mutations, p.Trp103Arg and p.Asp660His (Table 2), were reported to cause SPG54. Our results showed that three missense mutations including p.Val220Phe significantly reduced PLA_1_ activity (Figure 3), as predicted from the structural consideration (Figure 2). These data indicated that the loss of PLA_1_ activity significantly contributes to SPG54 pathogenicity. Furthermore, we demonstrated that the pVal220Phe mutant had marginal PLA_1_ activity (Figure 3A–C, lane 6). The result may account for the mild phenotype in the present case. The highly characteristic ^1^H-MRS finding of an abnormal lipid peak, which was also observed in our patient, has been considered to reflect accumulation of phospholipids as a result of abolished PLA_1_ activities. Although the p.Val220Phe mutation is not located in the SAM-DDHD domain, it is conceivable that the mutation associated with decreased PLA_1_ activity caused SPG54.

In conclusion, our report indicates that the possibility of SPG54 should also be taken into consideration when patients show a combination of adult-onset spastic ataxia and a thin corpus callosum. Magnetic resonance spectroscopy may be helpful in the differential diagnosis of patients with the spastic ataxia phenotype.

## Methods

### Patients

Clinical information, radiological images and blood samples were obtained from family members after written informed consent was provided. Experimental protocols were approved by the Independent Review Boards of Yokohama City University and Shinshu University. All experiments were performed in accordance with the institutional guidelines.

### Homozygosity mapping and exome sequencing

To identify the disease locus, genome-wide single nucleotide polymorphism (SNP) genotyping of the proband and the unaffected siblings was performed using the Genome-Wide Human SNP Array 6.0 (SNP 6.0 array) (Affymetrix, Inc., Santa Clara, CA). Then, SNP 6.0 array data were subjected to homozygosity mapping using HomozygosityMapper software^24^. The linkage analysis was performed based on the model of autosomal recessive inheritance with complete penetrance, using the subset of 7860 SNPs with high heterozygosity extracted from the SNP 6.0 array data with the program Linkdatagen, setting the bin size to 0.5 cM^25^. To find a gene mutation within the loci, whole exome sequencing was performed on the proband. The genomic DNA was processed using the SureSelect Human All Exon Kit v5 (Agilent Technologies, Santa Clara, CA).

### Structural consideration of an impact of the p.Val220Phe mutation in human DDHD2

A modeled structure of the region around Val220 in human DDHD2 was constructed from the crystal structure of the catalytic domain of guinea pig pancreatic lipase-related protein 2 (GPLRP2) chimerized with the C-terminal domain of human pancreatic lipase (HPL) (PDB id; 1GPL) using Phyre2 server^26^.

### Phospholipase A_1_ (PLA_1_) assay

The full-length *DDHD2* cDNA (FXC00119) was obtained from Kazusa DNA Research Institute (Chiba, Japan) and subcloned into pcDNA3.1/V5-His vector (Invitrogen). Site-directed mutagenesis was performed to produce *DDHD2* mutants with c.658G > T [p.Val220Phe], c.307T > C [p.Trp103Arg] and c.1978G > C [p.Asp660His] by using a mutagenesis kit (Toyobo, Osaka, Japan). All constructs were verified by Sanger sequencing.

The mammalian expression plasmid pEBG^27^ was used to express GST-fusion proteins. The WT and DDHD2 mutant cDNAs were inserted into pEBG and transfected into 293T cells using the Lipofectamine 2000 transfection reagent (Invitrogen) according to the manufacture's instruction. For a 3.5-cm culture dish, total 2 μg of the plasmids were used. Co-transfection of the WT- and each mutant-expressing constructs was performed in a 1:1 ratio. Their cellular lysates were prepared and mixed with Gluthathione Sepharose 4B beads (GE Healthcare, Piscataway, NJ), as described previously^23^. The lysates prepared from 293T cells grown on six 3.5-cm culture dishes were mixed with 22.5 μl of beads. After washing, half of the beads were used for PLA_1_ assay and the one-tenth was used for Western blotting analysis.

Preparation of ^32^P-labeled 1,2-dioleoyl-*sn*-phosphatidic acid (DOPA) and PLA_1_ assay were performed as described previously^23^. Briefly, reactions were started by adding 100 μl of a reaction buffer [50 mM Tris-HCl, pH 7.5, 100 mM KCl, 100 μM DOPA (Avanti Polar Lipids, Inc. Alabaster, AL) and 4.2 nM ^32^P-labeled DOPA to beads-containing tubes. The reactions were conducted at 37°C for 15 or 30 min, and then stopped by adding 200 μl of 1N HCl and 400 μl of CHCl_3_/methanol (2:1). The substrate (DOPA) and a product 2-oleoyl-lysophosphatidic acid (LPA) were recovered from an organic phase and separated by thin-layer chromatography (TLC). Radioactivity on a TLC plate was visualized using a Bio-Image Analyzer FLA-9000 (Fujifilm, Tokyo, Japan). For Western blotting analysis, an SDS sample buffer was added to the beads and the mixture was heated at 100°C for 5 min. The eluted proteins were analyzed by Western blotting, followed by staining with a rabbit polyclonal anti-GST antibody (Santa Cruz, CA).

## Supplementary Material

Supplementary Information Supplementary information

## Figures and Tables

**Table 1:** Homozygous SNVs detected in the proband

Gene	Frequency*	Mutation	SNP ID	SIFT score	PolyPhen2	Mutation Taster
***SPAG17***	0/575	c.2566T > G [p.Ser856Ala]		Tolerated, score 0.41	Possibly damaging, score 0.663	polymorphism
***SH3D19***	0/575	c.593A > G [p.Lys198Arg]		Tolerated, score 0.36	Possibly damaging, score 0.850	polymorphism
***DDHD2***	0/575	c.658G > T [p.Val220Phe]		**Affect protein function, score 0.00**	**Probably damaging, score 1.000**	**disease causing**
***ZNF169***	1/575	c.490T > C [p.Phe164Leu]	rs200089201	Tolerated, score 0.70	Benign, score 0.002	polymorphism
***ZNF462***	2/575	c.5650G > A [p.Gly1884Ser]	rs201673834	**Affect protein function, score 0.00**	Possibly damaging, score 0.702	**disease causing**
***FAM222A***	4/575	c.559C > T [p.Arg187Trp]	rs199694375	**Affect protein function, score 0.01**	**Probably damaging, score of 1.000**	**disease causing**
***GIT2***	3/575	c.1429C > A [p.Pro477Thr]	rs185965842	TOLERATED, score 0.29	Benign, score 0.200	**disease causing**
***NAA25***	1/575	c.564C > G [p.Asp188Glu]		Tolerated, score 0.52	Benign, score 0.024	**disease causing**

Only the SNVs, which were located within the candidate regions, unregistered in dbSNP137 or registered as uncommon SNPs with minor allele frequency <1%, and with the frequency <1% in exome data from 575 “in house” Japanese controls, are listed.

**Table 2:** Clinical features of SPG54 patients

Family (reference no.)	1^8^	2^8^	3^8^	4^8^	5^12^	6^12^	7^13^	8^14^	Current
*DDHD2* mutation(s)	p.Thr602Ilefs*18/p.Glu686Glyfs*35	p.Ile463Hisfs*6/p.Asp660His	p.Arg516*	p.Arg287*	p.Arg287*	p.Try661Cysfs*8	p.Asp660His	p.Trp103Arg/p.Asp660His	p.Val220Phe
	Clinical informations								
Consanguinity	−	−	+	+	+	−	−	−	+
Affected member(s)	two	two	five	one	two	two	Two	Two	one^a^
Age at examination	3 ~ 5	7 ~ 10	8 ~ 21	30	19 ~ 25	2 ~ 9	10 ~ 23	39–40	69
Age of onset (y.o.)	~2	~2	~2	~2	~6	0	0	4–5	45
Mental retardation	+	+	+	+	+	+	+	+	−
Optic-nerve hypoplasia	+	−	NA	+	NA	NA	−	NA	−
Strabismus	−	+	+	+	NA	NA	+	NA	−
Dysarthria	−	+	+	+	NA	NA	+	NA	+
Spasticity (upper/lower extremities)	−/+	+/+	±/+	−/+	−/+	−/+	+/+	NA	−/+
muscle weakness	−	+	+	+	NA	NA	+	+	+
Truncal ataxia	NA	NA	NA	NA	NA	NA	+	NA	+
Limb ataxia	NA	NA	NA	NA	NA	NA	+	NA	+
Extrapyramidal signs	−	±	−	+	−	−	−	NA	−
Involuntary movements	NA	NA	NA	NA	NA	NA	−	NA	postural tremor
Sensory involvements	NA	NA	NA	NA	vibratory sense	unknown	NA for vibratory sense	+	vibratory sense
Hyperreflexia	+	+	+	+	+	+	+	+	+
Extensor plantar responses	NA	NA	NA	NA	+	+	+	+	+
Peripheral neuropathy	NA	NA	NA	NA	−	−	mild decrease of MCV	+	mild decrease of SCV
Bladder disturbance	+	+	−	−	−	unknown	−	NA	+

Others					short stature	short stature			
	MRI/proton MRS findings								
Thin corpus callosum	+	+	+	+	+	+	+	+	+
White matter hyper intensities	+	+	+	+	NA	NA	+	+	±
Cerebellar atrophy	−	−	−	−	−	−	−	+	+
Syrinx	+	+	NA	−	NA	NA	NA	NA	NA
Abnormal lipid peak	+	+	+	+	NA	NA	NA	NA	+

Information regarding families 1 to 8 is from previous reports^8^,^12^,^13^,^14^. NA: not available.

^a^: one of two patients was genetically confirmed.
