# Supplementary material for: Late-onset spastic ataxia phenotype in a patient with a homozygous DDHD2 mutation
Source: Sci Rep. 2014 Nov 24;4:7132. doi: 10.1038/srep07132 (PMC5384088; doi:10.1038/srep07132)
Supplement: Supplementary Information [file srep07132-s1.pdf]

Supplementary Information

Title: Late-onset spastic ataxia phenotype in a patient with a homozygous *DDHD2* mutation

Authors: Hiroshi Doi,[1,2] Masao Ushiyama,[3] Takashi Baba,[4] Katsuko Tani,[4] Masaaki Shiina,[5] Kazuhiro Ogata,[5] Satoko Miyatake,[2] Yoko Fukuda Yuzawa,[6,7] Shoji Tsuji,[6] Mitsuko Nakashima,[2] Yoshinori Tsurusaki,[2] Noriko Miyake,[2] Hirotomo Saitsu,[2] Shu-ichi Ikeda,[8] Fumiaki Tanaka,[1] Naomichi Matsumoto[2] and Kunihiro Yoshida[9]

Affiliations:

[1]Department of Neurology and Stroke Medicine, [2] Human Genetics, and [5]Biochemistry, Graduate School of Medicine, Yokohama City University, 3-9 Fukuura, Kanazawa-ku, Yokohama 236-0004, Japan

[3]Department of Neurology, Kenwakai Hospital, 1936 Kanaenakadaira, Iida 395-8522, Japan

[4]School of Life Sciences, Tokyo University of Pharmacy and Life Sciences, 1432-1 Horinouchi, Hachioji, Tokyo, 192-0392, Japan

[6]Department of Neurology, Graduate School of Medicine, The University of Tokyo, 7-3-1 Hongo, Bunkyo-ku, Tokyo, 113-8655, Japan

[7]Max-Planck-Institute of Immunobiology and Epigenetics, Stübeweg 51, D-79108 Freiburg, Germany

[8]Department of Medicine (Neurology & Rheumatology), and [9]Division of Neurogenetics, Department of Brain Disease Research, Shinshu University School of Medicine, 3-1-1 Asahi, Matsumoto 390-8621, Japan

Correspondence:

Kunihiro Yoshida, kyoshida@shinshu-u.ac.jp

Division of Neurogenetics, Department of Brain Disease Research, Shinshu University School of Medicine, 3-1-1 Asahi, Matsumoto 390-8621, Japan

Supplementary figure legend

Figure S1. Intracellular distribution of DDHD2 in HEK293T cells was investigated. HEK293T cells in a 24-well plate were transfected with 0.5 μg of the pcDNA3.1/V5-His vector containing WT and p.Val220Phe mutant DDHD2 using the FuGENE^{R}HD transfection reagent (Roche Applied Science, Mannheim, Germany). Twenty four hours after transfection, cells were washed twice with ice-cold PBS, fixed for 20 min in 10% formalin/PBS, washed twice with PBS and permeabilized for 5 min with 0.1% Triton X-100/PBS. Double immunostaining using a rabbit polyclonal anti-V5 antibody (A190-120A, Bethyl Laboratories, Montgomery, TX) and a mouse monoclonal anti-GM130 (51-9001978, BD transduction laboratories, Franklin Lakes, NJ) were performed. A goat polyclonal anti-rabbit IgG conjugated to Alexa-488 (A-11008) and a goat polyclonal anti-mouse IgG conjugated to Alexa-546 (A-11030) were used as secondary antibodies (Molecular Probes, Eugene, OR), respectively. The anti-V5 antibody and the secondary antibodies were used at a 1:1000 dilution. The anti-GM130 antibody was used at a 1:500 dilution. WT and p.Val220Phe mutant DDHD2 diffusely distributed in cytoplasm with partial co-localization with Golgi-apparatus, which was consistent with previous report [1]. Bars = 10 μm.

Supplementary references

1 Baba, T., Yamamoto, A., Tagaya, M. & Tani, K. A lysophospholipid acyltransferase antagonist, CI-976, creates novel membrane tubules marked by intracellular phospholipase A1 KIAA0725p. *Mol Cell Biochem* **376**, 151-161, doi:10.1007/s11010-013-1563-4 (2013).

Figure S1

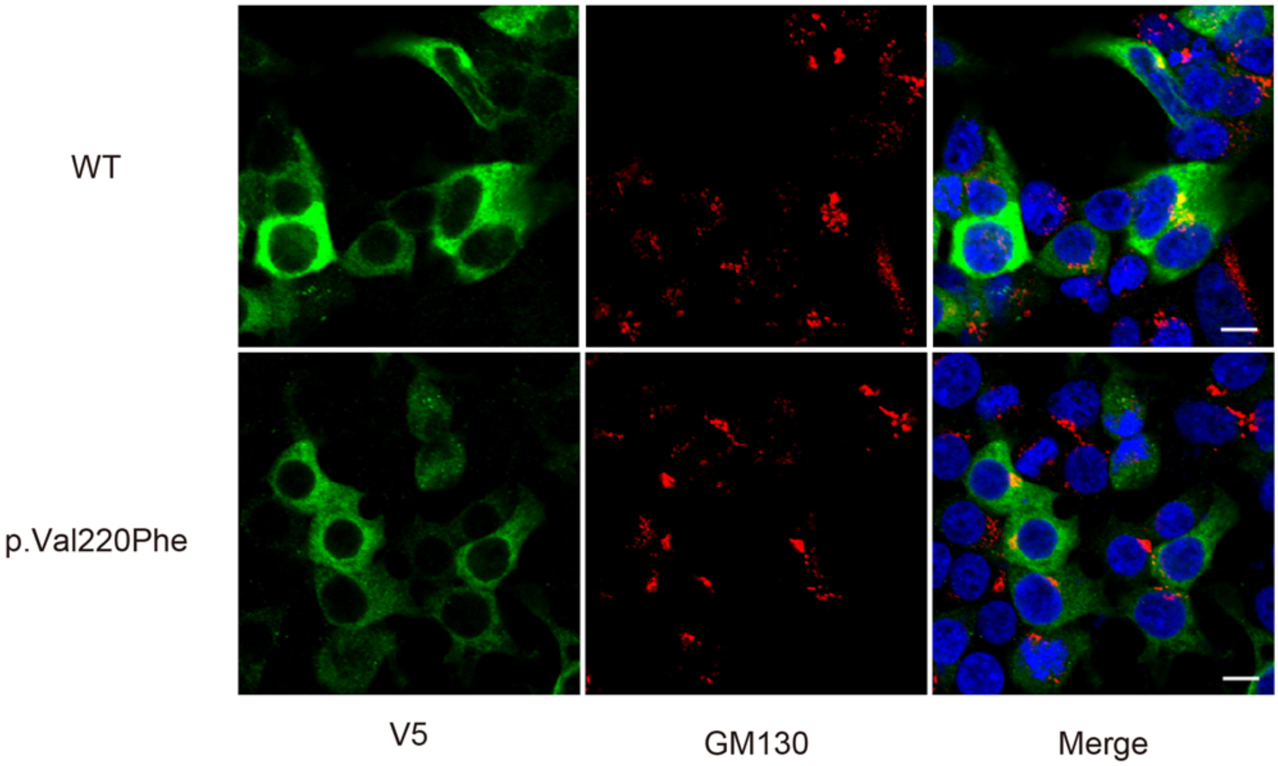

Table S1. Read coverage of ARCA and ARHSP genes

Disease	Gene	Covered by 10 or more reads
Friedrich ataxia	*FXN*	91.3
Ataxia with isolated vitamin E deficiency	*TTPA*	97.7
Ataxia, early-onset, with oculomotor apraxia and hypoalbuminemia	*APTX*	90.2
Abetalipoproteinemia	*MTTP*	100
Refsum disease	*PHYH*	100
	PEX7	100
Late-onset Tay-Sachs disease	*HEXA*	100
Late-onset Krabbe disease	*GALC*	99.3
Cerebrotendinous xanthomatosis	*CYP27A1*	97.3
DNA polymerase g disorders	*POLG*	100
Ataxia talangiectasia	*ATM*	98.9
Ataxia talangiectasia-like disorder	*MRE11B*	95.2
Autosomal recessive ataxia of Charlevoix-Saguenay	*SACS*	100
Mitochondrial DNA depletion syndrome 7	*C10orf2*	100
Cayman ataxia	*ATCAY*	100
Marinesco-Sjögren syndrome	*SIL1*	95.1
Sjögren-Larsson syndrome	*ALDH3A2*	100
SCAR1 (Ataxia-oculomotor apraxia 2)	*SETX*	100
SCAR5	*ZNF592*	100
SCAR8	*SYNE1*	99.9
SCAR9	*ADCK3*	100
SCAR10	*ANO10*	100
SCAR11	*SYT14*	96.9
SCAR12	*WWOX*	100
SCAR13	*GRM1*	100
SCAR14	*SPTBN2*	100
SCAR15	*KIAA0226*	99.8
SCAN1	*TDP1*	100
SPAX3, SPG70	*MARS2*	99.5
SPAX4	*MTPAP*	91.9
SPAX5	*AFG3L2*	92.1
Ataxia & Encephalopathy	*TTC19*	97.1
Ataxia with oculomotor apraxia 3 (AOA3)	*PIK3R5*	100
Dilated cardiomyopathy with ataxia	*DNAJC19*	100
Epilepsy, progressive myoclonic 6	*GOSR2*	98.4
Karak syndrome	*PLA2G6*	97
Myoclonus epilepsy of Unverricht-Lundborg	*CSTB*	100
Action myoclonus renal failure / Progressive myoclonus epilepsy without renal failure	*SCARB2*	100
Progressive myoclonus epilepsy-ataxia syndrome	*PRICKLE1*	100
Refsum-like disorder	*ABHD12*	100
Posterior column ataxia & Retinitis pigmentosa	*FLVCR1*	100

Seizures, sensorineural deafness, ataxia, mental retardation, electrolyte imbalance (SeSAME)	*KCNJ10*	100
α-Methylacyl-CoA racemase (AMACR) deficiency	*AMACR*	100
Niemann-Pick, Type C	*NPC1*	99.5
	NPC2	100
Wilson disease	*ATP7B*	99.1
SPG5A	*CYP7B1*	96.4
SPG7	*SPG7*	95.7
SPG11	*SPG11*	99.8
SPG15	*ZFYVE26*	98.3
SPG18	*ERLIN2*	100
SPG20	*SPG20*	98.3
SPG21	*SPG21*	100
SPG28	*DDHD1*	98.4
SPG30	*KIF1A*	99.2
SPG35	*FA2H*	93.1
SPG39	*PNPLA6*	99.3
SPG44	*GJC2*	79.9
SPG46	*GBA2*	99.5
SPG47	*AP4B1*	98.3
SPG48	*AP5Z1*	100
SPG49	*TECPR2*	100
SPG50	*AP4M1*	100
SPG51	*AP4E1*	100
SPG52	*AP4S1*	70.6
SPG53	*VPS37A*	87.1
SPG54	*DDHD2*	100
SPG55	*C12orf65*	100
SPG56	*CYP2U1*	93.2
SPG57, Hereditary motor sensory neuropathy-P (Okinawa type)	*TFG*	100
SPG58	*KIF1C*	100
SPG59	*USP8*	97.5
SPG60	*WDR48*	98
SPG61	*ARL6IP1*	97.4
SPG62	*ERLIN1*	100
SPG63	*AMPD2*	100
SPG64	*ENTPD1*	100
SPG66	*ARSI*	100
SPG67	*PGAP1*	100
SPG68	*FLRT1*	100
SPG69	*RAB3GAP2*	98
SPG71	*ZFR*	99.4
SPG72	*REEP2*	100
SPG73	*MAG*	100

The percentage of coding sequences covered by 10 or more reads.

Table S2 Regions identified by homozygosity mapping and multipoint linkage analysis in the model of autosomal recessive inheritance.

Chromosome	position	size (bp)
1	114,818,994-144,690,727	29,871,733
1	168,369,018-187,346,668	18,977,650
3	4,977,383-9,627,277	4,649,894
3	192,554,450-197,856,433	5,301,983
4	111,807,089-184,573,379	72,766,290
5	111,980,106-126,933,930	14,953,824
8	18,172,011-41,147,753	22,975,742
9	80,252,414-114,853,331	34,600,917
12	15,266,796-25,731,901	10,465,105
12	94,197,679-113,963,736	19,766,057
16	73,788,366-79,656,673	5,868,307
total		240,197,502

Table S3 The potentially compound heterozygous SNVs detected in the proband

Gene	Frequency*	Mutation	SNP ID	SIFT score	PolyPhen2	Mutation Taster
CEISR2	0/575	c.4951G>A [p.Gly1651Ser]	rs201428256	Tolerated with a score of 0.17	possibly damaging with a score of 0.901	**disease causing**
	0/575	c.7198C>A [p.Leu2400Ile]		Tolerated with a score of 0.39	benign with a score of 0.000	polymorphism
NBPF10	4/575	c.836T>G [p.Leu279Trp]	rs201965160	Tolerated with a score of 0.24	unanalyzable	polymorphism
	0/575	c.857T>C [p.Met286Thr]		Tolerated with a score of 0.05	unanalyzable	polymorphism
TTN	4/575	c.29938G>A [p.Ala9980Thr]	rs189286381	unanalyzable	**probably damaging with a score of 0.972**	polymorphism
	3/575	c.59849G>A [p.Arg19950Gln]		unanalyzable	**probably damaging with a score of 1.000**	**disease causing**
	1/575	c.96655G>A [p.Val32219Ile]		unanalyzable	**probably damaging with a score of 0.984**	**disease causing**
TDP2	0/575	c.982C>A [p.His328Asn]		Tolerated with a score of 0.48	benign with a score of 0.002	**disease causing**
	0/575	c.1028A>T [p.Asp343Val]		**Affect protein function with a score of 0.01**	possibly damaging with a score of 0.916	**disease causing**
INPPL1	1/575	c.3424C>A [p.Arg1142Ser]		Tolerated with a score of 0.15	benign with a score of 0.0	**disease causing**
	1/575	c.3491G>A [p.Arg1164Gln]	rs200679872	Tolerated with a score of 0.47	benign with a score of 0.355	**disease causing**
CBFA2T3	2/575	c.469A>G [p.Thr157Ala]	rs79103141	with a score of 0.05	benign with a score of 0.054	**disease causing**
	2/575	c.799C>G [p.Gln267Glu]	rs76244575	Tolerated with a score of 0.10	benign with a score of 0.000	**disease causing**
EPS8L1	3/575	c.1616G>A [p.Arg539Gln]	rs140692049	Tolerated with a score of 0.42	benign with a score of 0.005	polymorphism
	3/575	c.1957G>T [p.Val653Leu]	rs201428256	**Affect protein function with a score of 0.0**	**probably damaging with a score of 0.972**	**disease causing**

Only the SNVs, which were unregistered in dbSNP137 or registered as uncommon SNPs with minor allele frequency < 1%, and with the frequency < 1% in 575 “in house” Japanese control exome data are listed. More than two deleterious SNVs were detected only in *TTN,* that the defects are known to be the cause of cardiomyopathy.

*: Allele frequency in “in house” 575 Japanese control exome data.
